# Characterization of sympathicotonia in post‐covid condition (long covid) and healthy controls using long‐term electrodermal activity (EDA) follow‐up

**DOI:** 10.1111/cpf.70037

**Published:** 2025-11-23

**Authors:** Timo Mustonen, Pasi Kytölä, Hanna Lantto, Erika Lager, Velina Vangelova‐Korpinen, Hélène Virrantaus, Aleksandra Sulg, Sanna Stålnacke, Tatiana Posharina, Ritva Luukkonen, Arja Uusitalo, Päivi Piirilä, Mari Kanerva

**Affiliations:** ^1^ Department of Clinical Physiology Peijas Hospital, HUS Medical Diagnostic Center, Helsinki University Hospital and Helsinki University Helsinki Finland; ^2^ Faculty of Social Sciences Tampere University Tampere Finland; ^3^ Department of Clinical Physiology Park Hospital, HUS Medical Diagnostic Center, Helsinki University Hospital and Helsinki University Helsinki Finland; ^4^ Outpatient Clinic for Persistent Symptom Rehabilitation Helsinki University Hospital and Helsinki University Helsinki Finland; ^5^ Department of Statistics and Bioanalytics Finnish Institute of Occupational Health Helsinki Finland; ^6^ Division of Clinical Physiology and Nuclear Medicine HUS Medical Diagnostic Center, Helsinki University Hospital and Helsinki University Helsinki Finland; ^7^ Department of Internal Medicine and Rehabilitation Helsinki University Hospital and Helsinki University Helsinki Finland; ^8^ Department of Infection Control Turku University Hospital, The Wellbeing Services County of Southwest Finland Turku Finland

**Keywords:** ambulatory physiological monitoring, Autonomic nervous system, post‐acute COVID‐19 syndrome, skin conductance, sympathetic activation

## Abstract

**Purpose:**

After SARS‐CoV‐2 infection, some patients develop post‐COVID condition (PCC), often associated with sympathicotonia. This study aimed to characterize sympathicotonia in PCC patients using a novel long‐term electrodermal activity (EDA) analysis via a smart ring and evaluate its clinical applicability.

**Methods:**

Seventeen PCC patients were recruited from a Long Covid outpatient clinic, and 18 healthy controls volunteered. PCC patients were divided based on self‐reported symptoms into those with or without sympathicotonia. A 14‐day EDA monitoring was conducted. Sympathetic nervous system (SNS) activity was expressed as a double normalized index of electrodermal activity (DNE), with higher levels indicating higher SNS activity. Orthostatic tests were performed to identify orthostatic sympathicotonia. DNE levels, representing EDA, were compared to self‐reported and orthostatic sympathicotonia.

**Results:**

DNE levels did not differ between PCC patients with (*N* = 12) or without (*N* = 5) self‐reported sympathicotonia or compared with nonsympathetic controls. When dividing all participants by orthostatic test results, DNE levels were lower during day (08:00–14:00; *p* < 0.05) but higher during late night (00:00–02:00; *p* < 0.05) in those with orthostatic sympathicotonia (*N* = 21) compared to those without (*N* = 14), with the 24‐h comparison significant (*p* = 0.022). Among PCC patients, DNE levels were higher in orthostatic nonsympathicotonic (*N* = 7) than orthostatic sympathicotonic (*N* = 10) during morning (09:00–12:00; *p* < 0.05), with the 24‐h comparison significant (*p* = 0.044).

**Conclusion:**

Self‐reported symptoms did not distinguish sympathicotonia. However, individuals with orthostatic test‐identified sympathicotonia had heightened EDA, indicating increased sympathetic activity, particularly during late night. PCC was not identifiable by EDA. Long‐term EDA monitoring may provide an objective tool for detecting sympathicotonia independently of self‐reported symptoms.

## 1 INTRODUCTION

After acute SARS‐CoV‐2 infection, about 3%–10% (Alkodaymi et al., 2022; Ballering et al., 2022; Fernandez‐de‐las‐Peñas et al., 2024; Premraj et al., 2022) of patients develop variable symptoms, a condition called post‐COVID condition (PCC) or Long Covid (LC). This condition is diagnosed if no other diagnoses explain the symptoms which have lasted for at least 2 months and continue or develop at 3 months after the acute COVID‐19 infection (Havervall et al., 2021). PCC has been observed following both severe (Parotto et al., 2023) and mild COVID‐19 infections (Fernández‐de‐las‐Peñas et al., 2021). Several studies suggest that since the turn of the years 2021 and 2022, with the emergence of the Omicron variant, PCC has become less common, possibly due to the nature of the disease and increased vaccination coverage (Agergaard et al., 2023; Antonelli et al., 2022; Fernández‐de‐las‐Peñas et al., 2022). The symptoms of PCC include fatigue, exercise intolerance, cognitive disturbances, cough, dyspnea, chest pain, tachycardia, muscle pain, brain fog, depression and gastric symptoms (Fernández‐de‐las‐Peñas et al., 2021; Havervall et al., 2021; Parotto et al., 2023). Sympathetic overactivity, an imbalance between the sympathetic and parasympathetic functions (Aranyó et al., 2022; Barizien et al., 2021; Dani et al., 2021; Papadopoulou et al., 2022; Shouman et al., 2021), or even dysautonomia (Barizien et al., 2021; Ladlow et al., 2022; Papadopoulou et al., 2022) have been suggested as explanations for some of these symptoms, such as increased heart rates at rest and during and after physical activity (Inanc and Sabanoglu, 2022; Ladlow et al., 2022). Some studies have explained PCC as a multiorgan syndrome with vascular and/or cardiac complaints while others have interpreted the phenomenon from a biopsychosocial perspective (Raman et al., 2022; Rinaldo et al., 2021; Rudofker et al., 2022; Saunders et al., 2023). Although severe cardiac involvement, such as chronotropic incompetence (Aparisi et al., 2022; Durstenfeld et al., 2022; Mustonen et al., 2024; Szekely et al., 2021) has been reported, several extensive studies have not found any severe organic basis for the syndrome.

Among PCC patients, sympathetic activity has most commonly been measured using ECG‐based heart rate variability (Aranyó et al., 2022; Dani et al., 2021; Freire et al., 2023; Marques et al., 2023; Menezes Junior et al., 2022), sometimes also with the Ewing test battery or individual tests from the battery (Townsend et al., 2021). Other examples of methods used to study PCC symptoms include a microneurographic technique to measure muscle sympathetic activity and heart rate or blood pressure measurement with a finger photoplethysmography (Stute et al., 2022). Some researchers have also measured quantitative sudomotor axon reflex (Shouman et al., 2021). In some studies, electrodermal activity (EDA) has been used to assess sympathetic activity by studying sympathetic skin responses (SSRs) in laboratory settings (Bocci et al., 2023; Papadopoulou et al., 2022; Ser et al., 2022). However, as far as we know, EDA has not been previously utilized in long‐term follow‐up analysis of PCC patients in ambulatory settings. As known, the sympathetic nervous system (SNS) is activated during stress, leading to increased sweat production and enhanced electrical conductance of the skin. This physiological response results in heightened skin moisture content, which subsequently enhances the electrical conductivity of the skin's outer layer. EDA changes, therefore, serve as an indicator of SNS activity (Liu & Du, 2018).

Our aim was to analyze and assess the presence of sympathicotonia in PCC patients using (1) the presence of subjective symptoms, (2) objective hemodynamic responses measured during active orthostatic testing, and (3) an EDA follow‐up profile obtained with a new ambulatory method. We also aimed to examine whether the EDA profile differs between patients with self‐reported sympathicotonia and those without, in both patients and controls. Furthermore, we aimed to study whether the EDA profile differs according to orthostatic hemodynamic responses between sympathicotonic and non‐sympathicotonic participants. The aim was also to characterize the EDA patterns in both groups and to investigate the feasibility of EDA measurements for clinical use.

## 2 METHODS

### 2.1 Study design

**Figure 1:**
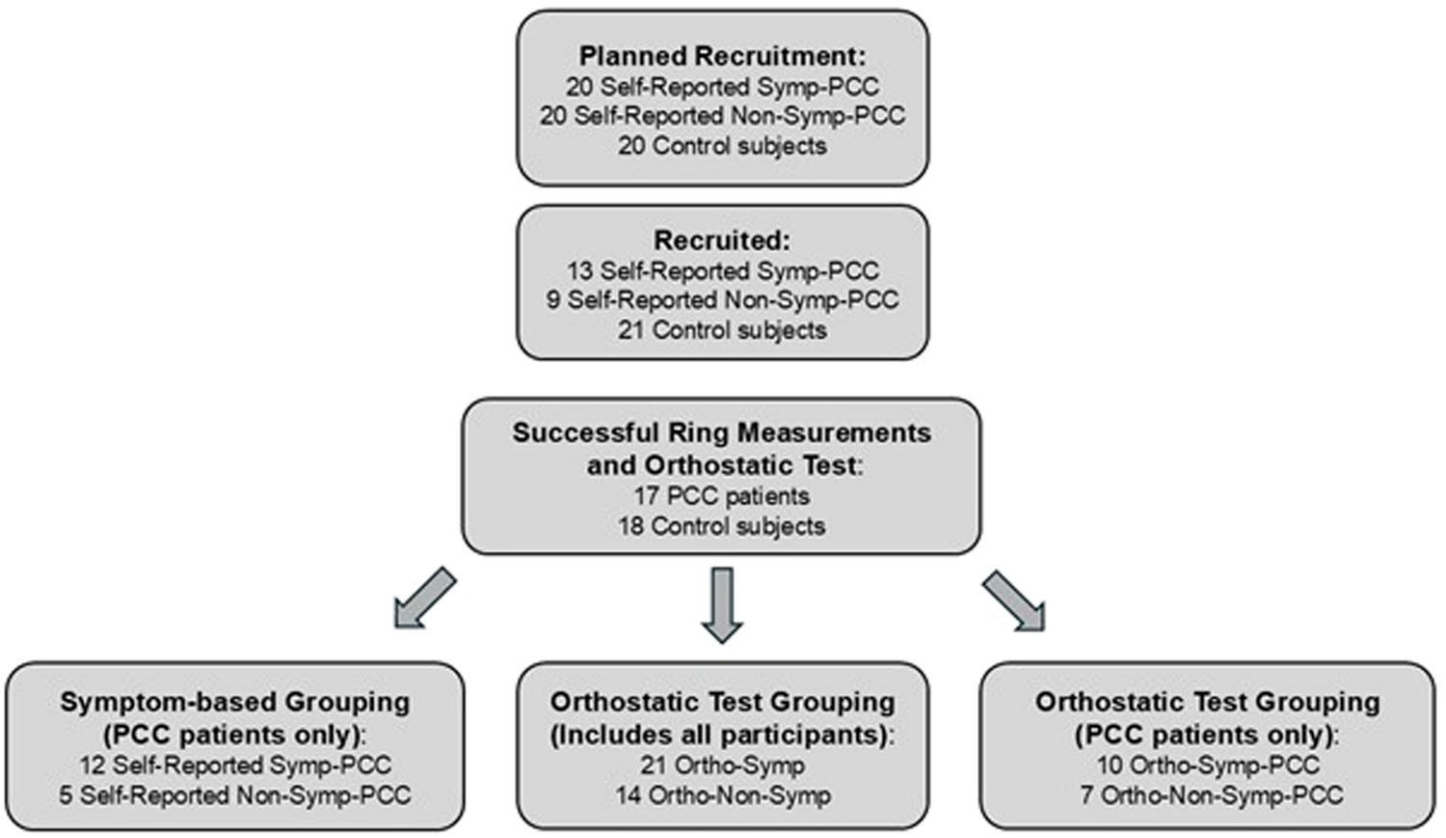
Study design for the selection and analysis of the patients and controls. ‘Self‐Reported Symp‐PCC’ = Post‐COVID condition patients who self‐reported symptoms suggestive of sympathicotonia. ‘Self‐Reported Non‐Symp‐PCC’ = Post‐COVID condition patients without self‐reported symptoms suggestive of sympathicotonia. ‘Ortho‐Symp’ = participants with a sympathicotonic orthostatic reaction. ‘Ortho‐Non‐Symp’ = participants without a sympathicotonic orthostatic reaction. ‘Ortho‐Symp‐PCC’ = Post‐COVID condition patients with a sympathicotonic orthostatic reaction. ‘Ortho‐Non‐Symp‐PCC’ = Post‐COVID condition patients without a sympathicotonic orthostatic reaction.

The study aimed to assess the presence or absence of sympathicotonia in patients with PCC symptoms. The study design is presented in Figure 1. The original goal was to recruit 20 patients who reported symptoms suggestive of sympathicotonia and 20 patients without such symptoms, as well as 20 healthy control subjects. However, it was challenging to find PCC patients without any symptoms suggestive of sympathicotonia, which is why the final numbers were lower than initially planned.

### 2.2 Patients

The patient group consisted of individuals who had been referred to the HUS LC outpatient clinic due to PCC symptoms (Table 1). Except for one patient who required a brief hospitalization without intubation, all others were treated at home during the acute phase. All patients underwent PCR testing, but the virus variant was not determined. The patients had COVID‐19 between March 2020 and December 2022, corresponding to the Alpha or Beta variant periods in two patients, and to the Omicron variants in the others (THL 2025). A total of 35 adult PCC patients were invited to participate in the study. They were recruited consecutively based on predefined inclusion criteria and their voluntary consent to participate. Patients who reported symptoms suggestive of sympathicotonia (e.g., a sensation of high resting heart rate, a feeling of excessive heart rate increase during exertion, palpitations, and/or sleep difficulties) were assigned to the sympathicotonic group, while those without these symptoms were placed in the non‐sympathicotonic group by the physicians or physiotherapists at the PCC outpatient clinic. Seventeen PCC patients completed successful ring follow‐ups (12 with self‐reported sympathicotonia and 5 without). The symptoms among patients without self‐reported sympathicotonia included fatigue (4 patients), depression (1), brain fog (2), dyspnea (1), decreased exercise tolerance or postexercise malaise (2), and neurological symptoms such as olfactory and taste changes, and possibly neurologically mediated muscle pain in the back or buttocks. Individual patients could present several symptoms. In the patients with self‐reported sympathicotonia, additional symptoms were as follows: fatigue (8 patients), exercise intolerance (4), brain fog (4), muscular or chest pain (5), neurological symptoms (4), sleep disturbance (2), dyspnea (1), and depression (1). Of all patients, 15 had received two to four vaccinations, and two had received none.

**Table 1:** Baseline characteristics and electrodermal activity results in PCC patients divided by self‐reported sympathicotonia, and in asymptomatic control subjects.

	PCC patients		Nonsymptomatic Control subjects *N* = 18	*p* value, ANOVA	*p* value, adjusted
	Self‐Reported Symp‐PCC *N* = 12	Self‐Reported Non‐Symp‐PCC *N* = 5			
Variable	Mean (SD)	Mean (SD)	Mean (SD)		
Sex, male/female	5/7	2/3	3/15	0.236^a^	‐
Age (y)	42.5 (9.9)	37.2 (6.0)	49.1 (10.9)	0.048	‐
BMI (kg/m^2^)	25.7 (3.8)	26.0 (4.8)	26.5 (4.4)	0.749	‐
COVID‐19 vaccinations before examinations (number)	2.9 (1.2)	3.7 (1.6)	2.4 (0.8)		
Unvaccinated (*n*)	1	1	1		
Time period of acute COVID‐19 infection	3/2020‐12/2022	6/2021‐6/2022	12/2021‐6/2022^b^		‐
Time from acute COVID‐19 infection to examinations (months)	13.7 (4.6)	15.8 (1.9)	11.9 (5.3)^b^	0.209	‐
Increased sympathetic activity in the orthostatic test (Thulesius criterion)	9 (75%)	2 (40%)	13 (72%)	‐	‐
Full day period DNE level	52.9 (6.1)	44.7 (13.6)	52.2 (3.7)	0.064	0.202
Night period (23:00‐07:00) DNE level	38.1 (14.3)	30.2 (15.2)	39.7 (9.4)	0.310	0.397
Day period (07:00‐23:00) DNE level	61.2 (8.4)	53.0 (17.8)	59.6 (6.9)	0.273	0.341
Late night (23:00‐03:00) DNE level	39.8 (14.8)	32.0 (12.7)	37.6 (10.6)	0.513	0.355
Early morning (03:00‐07:00) DNE level	36.4 (15.3)	28.3 (18.1)	41.9 (10.2)	0.130	0.877

*Note*: PCC patients were clinically divided into groups with increased or normal sympathetic activity based on self‐reported symptoms. Self‐Reported Symp‐PCC refers to PCC patients who reported symptoms suggestive of sympathicotonia (e.g., sensation of high resting heart rate, palpitations, or sleep disturbances), while Self‐Reported Non‐Symp‐PCC refers to PCC patients who did not report such symptoms. Healthy, non‐symptomatic control subjects are included for comparison. The presence of increased sympathetic activity was additionally analyzed according to the Thulesius (1976) criterion. DNE, a double normalized index of electrodermal activity; full day period DNE level, the average of daily DNE values across all 14 days; night period (23:00‐07:00) DNE level = the average of DNE values between 23:00 and 07:00 across all 14 days; day period (07:00‐23:00) DNE level = the average of DNE values between 07:00 and 23:00 across all 14 days; late night (23:00‐03:00) DNE level = the average of DNE values between 23:00 and 03:00 across all 14 days; early morning (03:00‐07:00) DNE level = the average of DNE values between 03:00 and 07:00 across all 14 days.

^a^ Fisher's exact test.

^b^ Control group included 8 participants with a history of COVID‐19.

As a part of the study protocol, patients wore smart rings for 14 days and underwent an active orthostatic test. Ring follow‐ups were conducted soon after the first visit to the LC outpatient clinic. Participation was not recommended for individuals with a nickel allergy due to the risk of an adverse skin reaction from wearing the smart ring. The investigations were carried out between October 5, 2022, and October 4, 2023 (Table 1).

### 2.3 Controls

The control group consisted of 21 healthy adults who did not have PCC symptoms and either had no history of COVID‐19 infection or had recovered from a PCR‐confirmed infection without developing PCC symptoms (*N *= 8). Both successful ring measurements and orthostatic test results were obtained from 18 controls (Table 1). The infections among the eight nonsymptomatic participants had occurred between November 2021 and March 2022, were treated at home, and took place during a period when Omicron variants were predominant. All but one control had received at least two SARS‐CoV‐2 vaccinations. Controls were hospital personnel (including some researchers) or their acquaintances who volunteered as a nonsymptomatic comparison group. They followed the same study protocol as the patient group, including 14 days of smart ring monitoring and an active orthostatic test.

The control subjects were also interviewed to rule out PCC‐like symptoms to confirm the eligibility as controls. They were additionally asked whether they had any symptoms suggestive of sympathicotonia. One participant without a history of COVID‐19 occasionally experienced mild sympathetic sensations, and another reported sleep difficulties and palpitations. Three participants had noticed a rapid increase in heart rate during exertion, two of them with a previous COVID‐19 infection and one without. One participant with a history of COVID‐19 reported sleep difficulties. These symptoms were described as unrelated to COVID‐19 infection or as having been present already before the infection.

## 3 MEASUREMENTS

### 3.1 Continuous and ambulatory electrodermal activity (EDA) measurement

The EDA measures were collected with Nuanic smart ring version V7AC_2 (formerly Moodmetric smart ring) (Nuanic Oy, Finland). The ring was primarily developed for continuous and ambulatory measurements, but it can be used to capture EDA data in two ways. For short‐term measurements it is possible to record raw EDA signal at a sampling rate of 3‐16 Hz. The signal accuracy of the ring has been investigated in two independent studies reaching an accuracy of 83% compared to that of a laboratory device (Pakarinen et al., 2019; Torniainen et al., 2015). EDA signals are prone to artifacts caused by movement, moisture and, other external factors (Boucsein, 2012). To enhance interpretability, Nuanic Oy has developed a proprietary parameter called the double‐normalized index of electrodermal activity (DNE). DNE provides a value between 1 and 100 every minute, allowing for continuous and ambulatory assessments of SNS activity. A higher DNE value indicates higher SNS activation. DNE was specifically designed to minimize the impact of confounding factors, making it particularly well‐suited for ambulatory settings (Salonius et al., 2024). DNE is calculated using a learning algorithm, which compares changes in EDA to the typical, individualized baseline values established while the participant wears the ring. During a 12‐h calibration period, an individual‐specific algorithm state is established to mitigate undesired influences, such as muscle movement, temperature fluctuations, and skin dryness.

Participants were instructed to wear the rings continuously for 2 weeks, except during showers. To ensure sufficient data quality, a minimum of four valid recording days was required, with at least 16 h of wear per day. Participants with fewer than four valid days were excluded from the study. The rings automatically collected DNE data, which participants uploaded twice daily to a cloud service via a mobile application. For each participant, hourly DNE values were averaged across all available measurement days at each hour. If a participant recorded data for the full 14‐day period, up to 840 min‐level values (14 × 60 per hour) were included in the calculation for each hour. Hourly differences between groups were analyzed using these pooled values to compare trends over time. The ring also includes a 3‐axis accelerometer that provides step count data. Participants maintained a daily diary to document activity levels, sensations of tension, stress, arousal, or anxiety, which were then compared to DNE values.

To summarize DNE levels and variability, six variables were created based on minute‐by‐minute observations. The daily DNE level was calculated for each 24‐h period, starting at 06:00, by averaging all minute‐by‐minute DNE values. The full‐day DNE level represents the average of daily DNE values across all 14 days, while the full‐day DNE variability represents the standard deviation of these values. The daytime DNE level was calculated as the average between 07:00 and 23:00, whereas the nighttime DNE level was determined from 23:00 to 07:00. Nighttime was further divided into late‐night (23:00–03:00) and early‐morning (03:00–07:00) periods for a more detailed analysis.

For quality assessment, number of successfully recorded days (at least 4 days with a minimum of 16 h per day) was considered for analysis. The average recording duration per day (minutes per day) and the average step count per day were also calculated. To assess physical activity levels, the step count per recorded minute was used as a measure.

### 3.2 Orthostatic test

Among PCC patients, active orthostatic tests were conducted approximately 1–2 months after the ring measurements (median 0.8 months in those with self‐reported sympathicotonia and 1.05 months in those without). Similarly, in the control group, the orthostatic tests were conducted approximately 1–2 months after EDA recording, following the same scheduling constraints as for PCC patients. The orthostatic tests in PCC patients were performed, on average, 14.1 months (SD 6.2; range 5–35) after the onset of a PCR‐positive COVID‐19 infection.

Participants were asked to refrain from smoking for 2 h and from drinking coffee or tea for 4 h before the examination. Two participants were using beta‐blockers; one discontinued the medication 2 days before the orthostatic test, following the laboratory's standard practice, while the other continued due to an arrhythmia condition. After a 10‐min rest in a supine position, blood pressure, heart rate, oxygen saturation and breathing frequency were recorded. A 12‐lead ECG was continuously monitored throughout the test, and measurements were recorded using computerized software (CardioSoft version 7‐7.0851; GE Medical Systems) alongside manual blood pressure measurements. The subject then stood up, and blood pressure was measured immediately upon standing and at 1‐min intervals thereafter. A continuous ECG was recorded throughout the test, with the highest heart noted immediately upon standing (approximately the 15th beat after standing up), followed by recordings at 1 min and at every full minute thereafter. The active standing phase lasted for a total of 8 min (Malmberg & Sovijärvi, 2000). A sympathicotonic orthostatic reaction was defined as a heart rate increase of ≥25 beats per minute, combined with either normal (±10 mmHg) or decreased systolic blood pressure compared to baseline, according to the criteria described by Thulesius (Thulesius, 1976).

### 3.3 Statistical methods

The data were analyzed using IBM SPSS Statistics version 27 and R software. Shapiro–Wilk's test was used to evaluate the normality of the outcome variables. A linear model was applied to assess the effects of self‐reported sympathicotonia, absence of sympathicotonia, and control status on DNE results, adjusting for age, sex, and body mass index (BMI). Differences between participants with and without sympathicotonia were assessed using non‐paired t‐tests, also adjusted for age, sex, and BMI.

To analyze the average hourly profile of DNE and physical activity (step counts) over a 24‐h period across study groups, linear mixed‐effects models were applied to assess the effect of group on hourly DNE and step count levels, adjusting for age, sex, and BMI. Estimated marginal means were calculated for each individual hour. Pairwise comparisons of means were performed, with *p*‐values adjusted using the Tukey method to control for family‐wise error rates. A bootstrap method was also applied to evaluate the overall average absolute difference between the estimated marginal means of two groups over a 24‐h period. *p*‐Values were computed as the proportion of null simulations that produced an absolute difference equal to or greater than the observed value.

Poisson regression with a log link was used to assess whether subjects with sympathicotonia, as determined by the orthostatic test, and late night (23:00–03:00) DNE levels above 35 reported more perceptions of tension, stress, arousal, or anxiety. The dependent variable was the proportion of diary days with reported perceptions. The model was adjusted for sex, age, and BMI. Estimated ratio means and 95% confidence intervals were calculated for both groups, with age, sex, and BMI standardized at their mean values.

A *p* < 0.05 was considered statistically significant.

## 4 RESULTS

Mean values for the number of valid measurement days across different groups are presented in Supporting Information S1: Tables S1–S3. The participants wore the ring for a median of 12 valid measurement days (range: 4–14 days) over a 2‐week period. Among the self‐reported groups, the duration of follow‐up was the shortest in the control group (*p* = 0.037), as shown in Supporting Information S1: Tables S1. Additionally, the total minutes recorded over 24 h, and specifically between 03:00 and 07:00, differed significantly among the groups (*p* = 0.026) (Supporting Information S1: Table S1). However, physical activity (=step counts/minute) and average step counts did not differ between the groups (Supporting Information S1: Tables S1 and S3).

### 4.1 Self‐reported sympathicotonia

**Figure 2:**
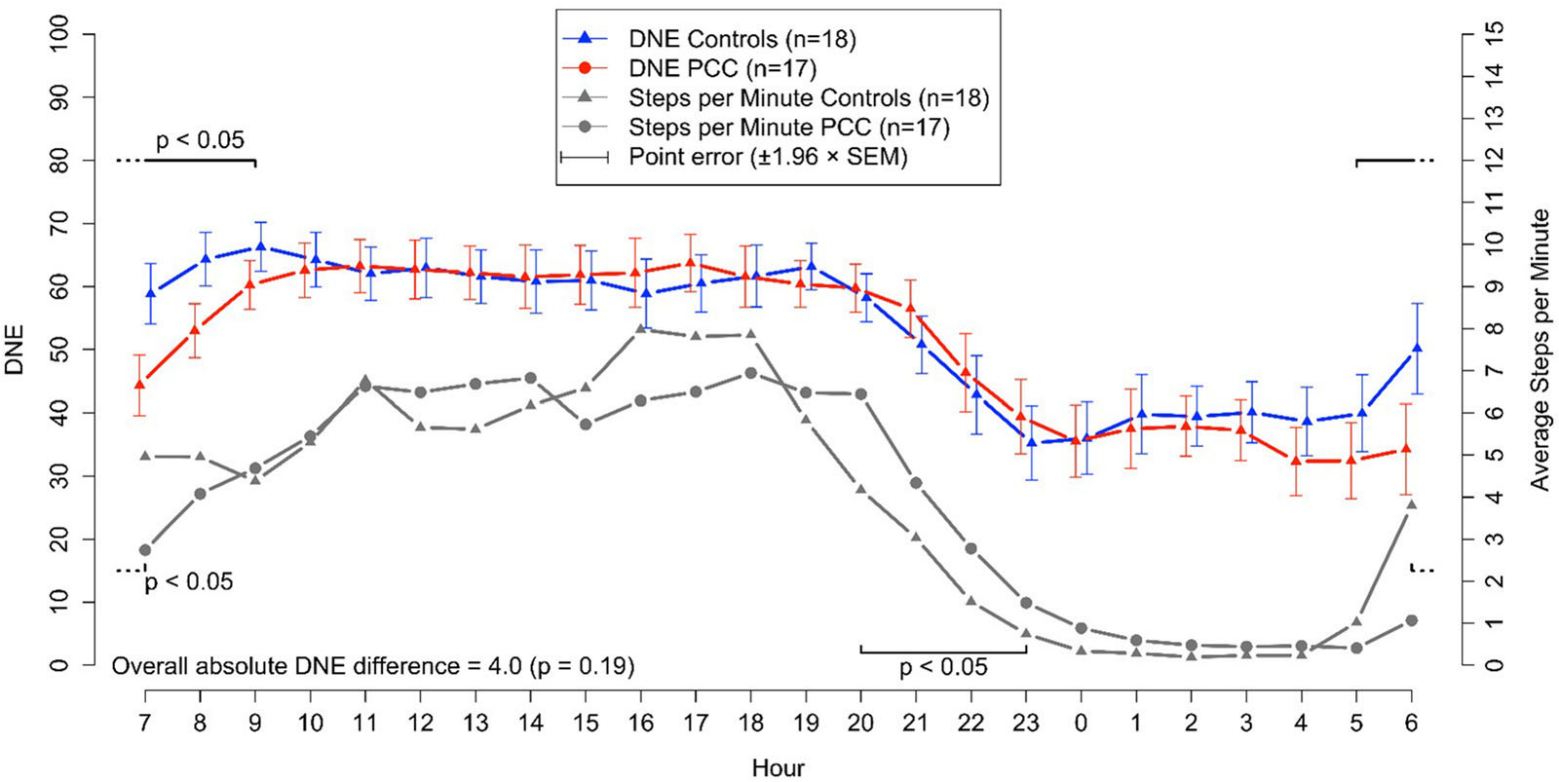
Longitudinal presentation and comparison of the mean DNE values over a 2‐week day and night follow‐up period between PCC patients (Self‐Reported Symp‐PCC + Self‐Reported Non‐Symp‐PCC) and control subjects. Simultaneous average step counts per minute are shown below the DNE curves. *p* values for significant hourly differences are shown just above or just below the respective curves, while the *p* value for the overall difference across the follow‐up period is shown at the bottom of the figure. A *p* < 0.05 was considered statistically significant. Error bars indicate the standard error of the mean for each hour, without accounting within‐subject correlations.

The mean DNE levels did not differ among the groups with self‐reported sympathicotonia, without sympathicotonia, and the controls (data not shown). In longitudinal analyses of self‐reported sympathicotonia, the 14‐day mean DNE (coverin both day and night periods) was 53 for PCC patients with self‐reported sympathicotonia (*N* = 12), 45 for those without self‐reported sympathicotonia (*N* = 5), and 52 for the control group (*N* = 18). These differences were not statistically significant (*p* = 0.11, data not shown). There were no significant differences in orthostatic test results between participants with and without self‐reported sympathicotonia (data not shown). However, in the longitudinal analysis between PCC patients (combining those with and without self‐reported sympathicotonia) and controls, the mean DNE level was higher in controls between 5:00 and 9:00 (*p* < 0.05). Nevertheless, the longitudinal difference between the groups was not significant when the entire 24‐h period was considered (*p* = 0.19; Figure 2). Physical activity (step counts/minutes) varied between groups, with PCC patients showing higher activity than controls between 20:00 and 23:00 (*p* < 0.05), but lower activity during the early morning (6:00–8:00, *p* < 0.05) and at noon (12:00–14:00, *p* < 0.05) (Figure 2).

### 4.2 Objectively measured sympathicotonia

Sympathicotonia, as determined by the orthostatic test, was observed in all three groups: patients with (9/12) and without self‐reported sympathicotonia (2/5), as well as the controls (13/18) (Table 1). Therefore, the data were further divided according to the orthostatic test results into those with a sympathicotonic orthostatic reaction (Ortho‐Symp, *N* = 21) and those without (Ortho‐Non‐Symp, *N* = 14), regardless of whether they were PCC patients or controls (Tables 1 and 2). Patients in the ortho‐Symp group were slightly leaner than those in the Ortho‐Non‐Symp patients (BMI 24.8 vs. 28.6; *p* = 0.006).

**Table 2:** Baseline characteristics and electrodermal activity results for all participants, including both PCC patients and control subjects, divided based on orthostatic test results indicating increased (Ortho‐Symp) or normal (Ortho‐Non‐Symp) sympathetic activity.

	Mean (SD)		Nonadjusted	Adjusted^a^
	Ortho‐Non‐Symp *n* = 14	Ortho‐Symp *n* = 21	*p*	*p*
Sex, male/female	4/10	6/15	0.652	‐
Age (y)	48.21 (10.7)	43.1 (10.5)	0.165	‐
BMI (kg/m^2^)	28.6 (4.0)	24.8 (3.61)	**0.006**	‐
Full day period DNE level	52.00 (3.51)	50.98 (8.60)	0.631	0.515
Night period (23:00‐07:00) DNE level	34.78 (10.09)	39.77 (13.23)	0.240	0.163
Day period (07:00‐23:00) DNE level	61.87 (5.27)	57.44 (11.34)	0.130	0.063
Late night (23:00‐03:00) DNE level	32.74 (9.65)	40.78 (13.06)	0.057	**0.046**
Early morning (03:00‐07:00) DNE level	36.99 (13.16)	38.80 (14.44)	0.709	0.453

*Note*: DNE = a double normalized index of electrodermal activity; full day period DNE level = the average of daily DNE values across all 14 days; night period (23:00‐07:00) DNE level = the average of DNE values between 23:00 and 07:00 across all 14 days; day period (07:00‐23:00) DNE level = the average of DNE values between 07:00 and 23:00 across all 14 days; late night (23:00‐03:00) DNE level = the average of DNE values between 23:00 and 03:00 across all 14 days; early morning (03:00‐07:00) DNE level = the average of DNE values between 03:00 and 07:00 across all 14 days. Bold values indicate statistically significant difference *p* < 0.05.

^a^ Adjusted for age, sex, and BMI.

**Figure 3:**
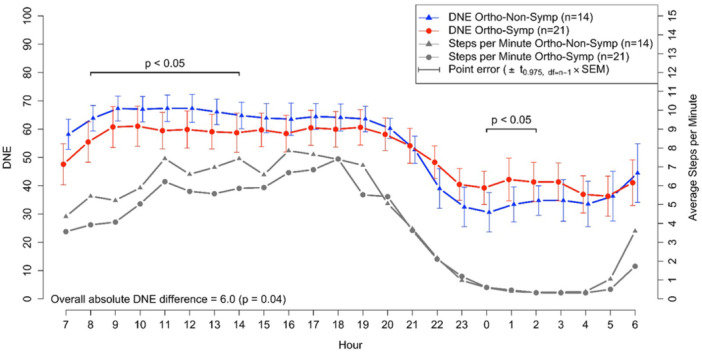
Longitudinal presentation and comparison of the mean DNE values over a 2‐week day and night follow‐up between Ortho‐Symp and Ortho‐Non‐Symp participants (*N* = 35). Simultaneous average step counts per minute are shown below the DNE curves. *p*‐Values for significant hourly differences are shown just above or just below the respective curves, while the *p*‐value for the overall difference across the follow‐up period is shown at the bottom of the figure. A *p* < 0.05 was considered statistically significant. Error bars indicate the standard error of the mean for each hour, without accounting within‐subject correlations.

In longitudinal analyses, when participants were divided according to orthostatic test results, daytime (07:00–23:00) DNE levels were numerically higher in the Ortho‐Non‐Symp group compared to the Ortho‐Symp group, 61.9 (5.3) versus 57.4 (11.3); however, this difference was not statistically significant (*p* = 0.063). In contrast, during the late‐night period (23:00–03:00), DNE levels were significantly higher in the Ortho‐Symp group compared to the Ortho‐Non‐Symp group, 40.8 (13.1) versus 32.7 (9.7); *p* = 0.046 (Table 2). In the longitudinal analysis, the Ortho‐Non‐Symp group had higher DNE levels between 8:00 and 14:00, while the Ortho‐Symp group had higher DNE levels between 00:00 and 02:00. Additionally, the DNE difference across the entire day and night period was significant (*p* = 0.04) (Figure 3).

Participants in the Ortho‐Symp group with late night DNE levels above 35 reported feelings of tension, stress, arousal, or anxiety on approximately one‐third (0.33, 95% CI: 0.24, 0.45) of the days, compared to about one‐quarter (0.23, 95% CI: 0.18, 0.30) of the days in the Ortho‐Non‐Symp group according to the daily forms completed during follow‐up.

### 4.3 Analysis among PCC patients

Based on orthostatic test results, PCC patients were divided into Ortho‐Symp‐PCC (*N* = 10) and Ortho‐Non‐Symp‐PCC (*N* = 7) groups, while control subjects were similarly classified into Ortho‐Symp‐Cont (*N* = 11) and Ortho‐Non‐Symp‐Cont (*N* = 7) groups. The number of recording days and total recorded minutes varied between groups classified according to the orthostatic test, but average step counts and physical activity levels did not differ (Supporting Information S1: Tables S3 and S4).

**Figure 4:**
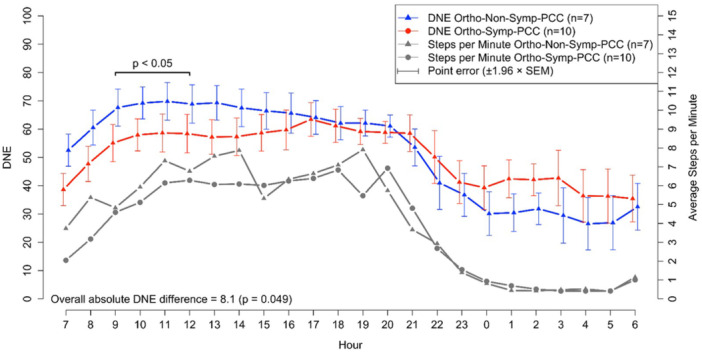
Longitudinal presentation and comparison of the mean DNE values over a 2‐week day and night follow‐up between Ortho‐Symp‐PCC and Ortho‐Non‐Symp‐PCC patients. Simultaneous average step counts per minute are shown below the DNE curves. *p*‐Values for significant hourly differences are shown just above the curves, while the *p*‐value for the overall difference across the follow‐up period is shown at the bottom of the figure. A *p* < 0.05 was considered statistically significant. Error bars indicate the standard error of the mean for each hour, without accounting within‐subject correlations.

When results for PCC patients only were analyzed, mean DNE levels during the late‐night period (23:00–03:00) were numerically higher in those with sympathicotonia (Ortho‐Symp‐PCC, DNE 41.2 ± 16.9) compared to those without (Ortho‐Non‐Symp‐PCC, DNE 32.2 ± 7.8), but this difference was not statistically significant (*p *= 0.091; Table 3). In the longitudinal analysis, between 9:00 and 12:00, DNE levels were significantly higher in the Ortho‐Non‐Symp‐PCC group compared to the Ortho‐Symp‐PCC group. Although a visual inspection suggested higher DNE levels in the Ortho‐Symp‐PCC group between 1:00 and 5:00, the difference compared to Ortho‐Non‐Symp was not statistically significant. However, the overall DNE difference between the groups over the full 24‐h period was significant (*p* = 0.049; Figure 4). Simultaneous step counts (steps per minute) did not differ between the groups.

**Table 3:** Baseline characteristics and electrodermal activity (EDA) results for PCC patients divided into groups, based on orthostatic test results indicating sympathicotonia (Ortho‐Symp‐PCC) or normal sympathetic activity (Ortho‐Non‐Symp‐PCC).

	Mean (SD)		Nonadjusted	Adjusted^a^
	Ortho‐Non‐Symp‐PCC *n* = 7	Ortho‐Symp‐PCC *n* = 10	*p*	*p*
Sex, male/female	3/4	4/6	0.646	‐
Age (y)	44.9 (8.2)	38.2 (9.0)	0.141	‐
BMI (kg/m^2^)	28.6 (3.6)	23.8 (2.9)	**0.008**	‐
Time from acute COVID‐19 infection to examinations (months)	14.83 (3.5)	14.11 (4.4)	0.744	‐
Full day period DNE level	51.23 (1.98)	50.02 (12.30)	0.767	0.956
Night period (23:00‐07:00) DNE level	30.50 (8.96)	39.42 (16.96)	0.225	0.087
Day period (07:00‐23:00) DNE level	62.65 (5.78)	56.13 (14.54)	0.223	0.307
Late night (23:00‐03:00) DNE level	32.17 (7.79)	41.22 (16.89)	0.209	0.091
Early morning (03:00‐07:00) DNE level	28.79 (11.82)	37.72 (18.12)	0.272	0.101

*Note*: DNE = a double normalized index of electrodermal activity; full day period DNE level = the average of daily DNE values across all 14 days; night period (23:00‐07:00) DNE level = the average of DNE values between 23:00 and 07:00 across all 14 days; day period (07:00‐23:00) DNE level = the average of DNE values between 07:00 and 23:00 across all 14 days; late night (23:00‐03:00) DNE level = the average of DNE values between 23:00 and 03:00 across all 14 days; early morning (03:00‐07:00) DNE level = the average of DNE values between 03:00 and 07:00 across all 14 days. Bold value indicates statistically significant difference *p* < 0.05.

^a^ Adjusted for age, sex and BMI.

### 4.4 Analysis among controls

When the control group was analyzed separately, no significant differences in DNE levels were observed between those with and without sympathicotonia (data not shown).

## 5 DISCUSSION

In the present study, sympathicotonia based on participants' self‐reported symptoms did not correspond well to orthostatic test‐identified sympathicotonia or to DNE levels during long‐term monitoring. When the mixed cohort of PCC patients and control participants was divided according to orthostatic test results, elevated DNE levels indicating sympathicotonia were most evident during the late‐night period. Simultaneous time‐related step counts did not differ between sympathicotonic and nonsympathicotonic participants, supporting the interpretation that increased DNE levels were likely due to sympathetic activation rather than physical activity. This pattern was similar in PCC patients, whereas no clear pattern emerged in controls. These observations represent novel findings.

Sleeping time represents a relatively stable state both physically and mentally, making it reliable and easy to interpret. However, in stress and disease states, sleep is often disturbed (Jacob et al., 2022; Park & Oh, 2023; Siddique et al., 2021). The late‐night increase in DNE observed in our study may reflect difficulties in falling asleep or prolonged stress interfering with normal relaxation during sleep. Alternatively, the findings might indicate altered autonomic regulation, which could contribute to sleep disturbances and non‐restorative sleep. To our knowledge, no previous studies have examined differences in EDA between late‐night and early‐morning periods in the context of sympathicotonia. While our study did not include direct sleep assessments, the results suggest that EDA measurements based on the DNE index could provide insights into autonomic activity during nighttime, offering potential applications for sleep analysis in future research.

Previous studies have shown that general DNE levels are higher during the daytime and lower during the nighttime in normal ambulatory EDA monitoring (Salonius et al., 2024). This pattern supports the idea of individuals' normal transition from wakefulness to sleep, when sympathetic tone decreases representing typical circadian rhythm patterns (Cardinali, 2018; Doberenz et al., 2011; Salonius et al., 2024; Somers et al., 1993). Variations in daily autonomous nervous function have been found to be associated with stress (Brugnera et al., 2019; Schiweck et al., 2021), and the combination of stress and depressive symptoms leads to a blunted heart rate circadian rhythm (Lutin et al., 2022). According to a recent study, individuals classified as healthy but exhibiting varying levels of depressive symptoms may show atypical cardiovascular responses to stressful events (Brugnera et al., 2019). Patients with PCC often present with depression (Cázares‐Lara et al., 2025). In earlier studies, increased heart rate with reduced variation, and lower heart rate variability have been observed in individuals with depression during both daytime and nighttime (Schiweck et al., 2021). This variation is most probably due to a decrease in activity of parasympathetic modulation of cardiac function (Saad et al., 2020). However, depressive symptoms were not assessed in this study.

Sympathicotonia is considered a physiological phenomenon that can occur even in healthy individuals as part of normal autonomic regulation. However, prolonged or excessive sympathetic activation has been implicated in various autonomic dysfunctions. Several studies on PCC have reported increased sympathetic activity, dysautonomia (Aranyó et al., 2022; Barizien et al., 2021; Dani et al., 2021; Ladlow et al., 2022; Papadopoulou et al., 2022; Shouman et al., 2021; Zanoli et al., 2022) or postural orthostatic tachycardia syndrome (POTS)‐like condition (Seeley et al., 2023). Saunders et al. (2023) propose a biopsychosocial model for explaining PCC, suggesting that in the presence of predisposing factors, COVID‐19 infection may trigger the brain's stress systems (Kozlowska, 2020), leading to sympathetic activation and changes in hormonal and inflammatory regulation. Prolongation of these changes has been hypothesized to cause sensitization of the central nervous system, contributing to the persistence of the symptoms (Goudman et al., 2021). Previous research has speculated that prolonged inflammatory responses triggered during the acute phase could lead to endotheliopathy, mitochondrial dysfunction, vascular stiffness, vascular injury, and cardiovascular complications (Aranyó et al., 2022; Araújo et al., 2023; Karakasis et al., 2024; Marques et al., 2023; Saeed et al., 2021). It is not known, however, whether these changes could persist and contribute to post‐acute syndromes. Arterial stiffness and sympathetic overactivity have been shown to be reversible phenomena (Zanoli et al., 2022). PCC symptoms, including those suggestive of increased sympathetic activity, can develop after both mild and severe acute COVID‐19 disease (Barizien et al., 2021; Fernández‐de‐las‐Peñas et al., 2021; Havervall et al., 2021; Parotto et al., 2023).

The present study adopted Thulesius' criteria for sympathicotonic heart rate and blood pressure changes (Thulesius, 1976). These criteria define a heart rate increase of ≥25 bpm, which is lower than the POTS threshold (≥30 bpm) and transient, in contrast to the POTS definition of sustained tachycardia (Raj et al., 2020). Our findings suggest that characterizing intermediate cases of sympathicotonia—those falling between normal autonomic responses and POTS—may be valuable, even though these cases are not as clinically pronounced as POTS. Our clinical observations suggest that sympathicotonia may represent an inherent personal trait or autonomic sensitivity rather than a pathological condition. Based on our findings, we suggest that even individuals with milder, transient orthostatic changes may warrant further study, as this group could provide new insights into autonomic nervous system function and variability.

The present study demonstrates the uncertainty associated with self‐reported sympathicotonia compared with objectively measured sympathetic reactions in the orthostatic test. Subjective factors, such as altered interoception or anxiety related to PCC symptoms, may have influenced the self‐reporting during outpatient visits (Chen et al., 2021; Henningsen et al., 2018). Patients may also have been particularly aware of PCC symptoms as presented by the media during and immediately after the years of the COVID‐19 pandemic, which could have influenced their self‐reports. Furthermore, the present results suggest that sympathicotonia may be very common and not necessarily associated with any disease conditions. Pakarinen et al. (2019) found that EDA was more accurate in classifying stress levels compared to self‐report questionnaires, suggesting that individuals may have difficulty in accurately recognizing and reporting their own stress levels. Additionally, only seven (29%) of the participants reported symptoms consistent with sympathicotonia during the sympathetic reaction in orthostatic test, highlighting the difficulty in recognizing sympathetic symptoms. The results confirm that increased sympathetic activity is not present in all PCC patients, and self‐reported symptoms may not reliably reflect physiological sympathicotonia. The findings suggest that EDA measured via the DNE index may serve as a useful method for detecting and analyzing sympathicotonia in PCC.

The control group, as well as the Ortho‐Non‐Symp and Ortho‐Non‐Symp‐PCC groups, exhibited higher DNE values during early morning and daytime compared to the groups with sympathicotonia. This difference likely reflects normal sympathetic function, where higher EDA levels are associated with increased physical activity, as also indicated by step counts. While these patterns may partly be explained by differences in daily routines, such as morning exercise or commuting to work, participant diaries were too inconsistent to reliably determine individual activity levels at different times of the day.

In the present study, the patients had experienced mild COVID‐19, and thorough examinations in the outpatient clinic did not reveal specific complaints related to the prior infection. However, increased DNE levels were observed in individuals with orthostatic sympathicotonia. EDA primarily reflects direct sympathetic activation at the skin level, whereas heart rate variability (HRV) is often used as an indirect measure of autonomic balance, with reduced HRV typically associated with increased sympathetic dominance. Previous studies have shown that autonomic nervous system dynamics, including sympathetic activation, play a role in sleep regulation (Peplow, 2013; Zapalac et al., 2024). Although our study did not include direct assessments of sleep stages, the ability of the DNE index‐based EDA measurement to capture nocturnal sympathetic activity makes it a valuable tool for understanding autonomic function during sleep.

One of the key advantages of the DNE index is its ability to assess and monitor sympathicotonia independently of self‐reported symptoms. Unlike subjective symptom assessments, which may be influenced by individual perception and variability, the DNE index provides an objective, continuous measure of sympathetic nervous system activity. This is particularly relevant in the context of PCC, where autonomic dysfunction may contribute to various symptoms. Monitoring based on the DNE index could aid in identifying the physiological basis of these symptoms and offer a tool for tracking autonomic regulation over time. In clinical practice, the ability to quantify sympathicotonia using EDA may help optimize symptom management and guide rehabilitation strategies. Further studies are warranted to explore its potential applications in personalized treatment approaches for PCC patients.

An interesting aspect of the present study lies not only in how the smart ring device detects sympathicotonia, but also in how a single orthostatic test can effectively capture signs of sympathicotonia. Despite the relatively small number of patients, the findings related to sympathicotonia were consistent and logically interpretable.

### 5.1 Strengths and limitations

The study presents a longitudinal clinical follow‐up using a novel method based on EDA measurement. The EDA recordings were of high quality, and participants wore the ring for a sufficient duration to allow reliable comparisons. The initial plan was to divide participants based on self‐reported symptoms of sympathicotonia. However, the study highlighted the difficulty of accurately self‐recognizing this condition. In contrast, the orthostatic test provided an objective means to detect sympathicotonia. Nevertheless, the number of participants was relatively small, which may limit the generalizability of the findings. Despite this, the results are logical and suggest that sympathicotonia was reliably assessed.

Eight women in our study used either hormonal contraception (*n* = 3) or hormone replacement therapy (*n* = 5). It is known that sex hormones may influence skin conductivity (White & Graham, 2016). We also added hormonal therapy to the model, but the model showed instability due to the strong correlation between gender and hormonal therapy. Therefore, hormonal therapy was not included as an adjustment factor in our final analyses. However, the influence of hormonal therapy on EDA values warrants further study with larger patient cohorts.

Another limitation is related to the diary recordings. Although participants were asked to fill out a diary detailing their daily activities and sleep periods, the entries varied considerably in quality and completeness. Therefore, reliable data on sleep duration and quality were not available. Step counts, however, provided valuable insights into participants' activity levels.

Additionally, there was a 1–2‐month delay between the EDA monitoring period and orthostatic testing for both patients and controls due to laboratory scheduling constraints. This delay did not significantly affect the results, as it was similar for both groups. Moreover, an individual's tendency toward sympathicotonia may be partly determined by inherent personal characteristics or sensitivities, making short‐term delays less likely to alter their sympathicotonia response in this kind of time delay.

## 6 CONCLUSION

Self‐reported presence or absence of sympathicotonia did not correspond to sympathicotonia detected objectively by orthostatic testing or measured using the new DNE‐based electrodermal activity method in either PCC patients or control subjects. However, in individuals with orthostatic sympathicotonia, DNE values were elevated during the late night, consistent with electrodermal sympathicotonia. The DNE analysis did not distinguish PCC patients from controls. Nevertheless, the results suggest that the DNE index is a useful objective method for detecting sympathicotonia in both healthy subjects and PCC patients, even in cases where individuals do not subjectively perceive symptoms related to increased autonomic nervous activity.

## AUTHOR CONTRIBUTIONS

Timo Mustonen, Päivi Piirilä and Mari Kanerva conceived the study; Timo Mustonen, Pasi Kytölä, Ritva Luukkonen and Päivi Piirilä analyzed data; Timo Mustonen, Pasi Kytölä and Päivi Piirilä drafted manuscript; Timo Mustonen and Pasi Kytölä prepared the figures; Päivi Piirilä and Mari Kanerva acquired funding; Päivi Piirilä, Mari Kanerva and Arja Uusitalo supervised the study and provided resources; Timo Mustonen, Pasi Kytölä, Hanna Lantto, Erika Lager, VV‐L, Hélène Virrantaus, Aleksandra Sulg, Sanna Stålnacke, Tatiana Posharina, Ritva Luukkonen, Arja Uusitalo, Päivi Piirilä and Mari Kanerva edited and revised manuscript. Timo Mustonen, Pasi Kytölä, Arja Uusitalo, Päivi Piirilä and Mari Kanerva approved final version of manuscript. All authors meet the ICMJE criteria for authorship.

## CONFLICT OF INTEREST STATEMENT

The authors declare no conflicts of interest.

## Supporting information

Table S1. Medication use and DNE recording quality data for groups, based on self‐reported increased sympathetic activity (Self‐Reported Symp‐PCC), self‐reported normal sympathetic activity (Self‐Reported Non‐Symp‐PCC), and non‐symptomatic control subjects. Table S2. Orthostatic test results, medication use, and DNE recording quality data for groups, classified based on orthostatic sympathetic response (Ortho‐Symp and Ortho‐Non‐Symp). Table S3. Baseline characteristics, medication use, DNE values, and DNE recording quality data for PCC patients and controls divided into four groups, based on orthostatic sympathetic response (Ortho‐Non‐Symp‐Control, Ortho‐Symp‐Control, Ortho‐Non‐Symp‐PCC, Ortho‐Symp‐PCC).

## Data Availability

Data is provided within the manuscript or supplementary information files. The data is not publicly available due to privacy statements.
