# Supplementary material for: Characterization of sympathicotonia in post‐covid condition (long covid) and healthy controls using long‐term electrodermal activity (EDA) follow‐up
Source: Clin Physiol Funct Imaging. 2025 Nov 23;45(6):e70037. doi: 10.1111/cpf.70037 (PMC12641159; doi:10.1111/cpf.70037)
Supplement: Supplementary file 1 — Table S1. Medication use and DNE recording quality data for groups, based on self‐reported increased sympathetic activity (Self‐Reported Symp‐PCC), self‐reported normal sympathetic activity (Self‐Reported Non‐Symp‐PCC), and non‐symptomatic control subjects. Table S2. Orthostatic test results, medication use, and DNE recording quality data for groups, classified based on orthostatic sympathetic response (Ortho‐Symp and Ortho‐Non‐Symp). Table S3. Baseline characteristics, medication use, DNE values, and DNE recording quality data for PCC patients and controls divided into four groups, based on orthostatic sympathetic response (Ortho‐Non‐Symp‐Control, Ortho‐Symp‐Control, Ortho‐Non‐Symp‐PCC, Ortho‐Symp‐PCC). [file CPF-45-0-s001.docx]

Table S1. Medication use and DNE recording quality data for groups, based on self-reported increased sympathetic activity (Self-Reported Symp-PCC), self-reported normal sympathetic activity (Self-Reported Non-Symp-PCC), and non-symptomatic control subjects.

|  | **PCC Patients** | | **Non-symptomatic**  **control subjects**  **N = 18** | **p-value,**  **ANOVA** | **p-value, adjusted** |
| --- | --- | --- | --- | --- | --- |
|  | **Self-Reported Symp-PCC**  **N = 12** | **Self-Reported**  **Non-Symp-PCC**  **N = 5** |  |  |  |
| Variable | Mean (SD) | Mean (SD) | Mean (SD) |  |  |
|  |  |  |  |  |  |
| **Medication** |  |  |  |  |  |
| Asthma medication | 2 | 3 | 1 |  |  |
| Allergy medication | 2 | 1 |  |  |  |
| Antihypertensive medication |  |  | 1 |  |  |
| Beta blockers | 1 | 1 |  |  |  |
| Hormone replacement therapy or contraceptives | 3 | 1 | 4 |  |  |
| Tranquilizers | 4 | 1 | 3 |  |  |
| Sleeping pills | 1 |  |  |  |  |
| Melatonin | 3 |  |  |  |  |
| Hypercholesterolemia medication | 1 | 1 | 1 |  |  |
| Anti-inflammatory medication | 1 |  |  |  |  |
|  |  |  |  |  |  |
| **DNE recording quality** |  |  |  |  |  |
| **Full day and night period** |  |  |  |  |  |
| Number of days | 11.8 (2.0) | 12.4 (1.8) | 10.3 (2.7) | 0.130 | **0.037** |
| Physical activity (step counts/min) | 3.8 (1.24) | 4.0 (1.51) | 3.8 (1.24) | 0.759 | 0.637 |
| Average minutes | 1321.7 (62.6) | 1309.8 (38.9) | 1252.3 (73.1) | **0.021** | **0.038** |
| Average step counts | 5043.1 (1660.2) | 5228.4 (2090.7) | 4738.7 (1644.2) | 0.809 | 0.442 |
| **Night period (23:00-07:00)** |  |  |  |  |  |
| Physical activity (step counts/min) | 0.78 (0.71) | 0.56 (0.32) | 0.81 (0.73) | 0.467 | 0.898 |
| Average minutes | 462.7 (12.4) | 464.4 (11.3) | 453.7 (15.7) | 0.154 | 0.248 |
| Average step counts | 356.4 (318.4) | 257.8 (147.6) | 369.0 (338.3) | 0.779 | 0.902 |
| **Day period (07:00-23:00)** |  |  |  |  |  |
| Physical activity (step counts/min) | 5.46 (1.97) | 5.85 (2.20) | 5.5 (1.81) | 0.695 | 0.681 |
| Average minutes | 858.8 (55.9) | 845.6 (36.4) | 788.6 (73.1) | **0.043** | 0.068 |
| Average step counts | 4686.5 (1705.2) | 4970.6 (1994.3) | 4369.8 (1505.0) | 0.743 | 0.409 |
| **Late night (23:00-03:00)** |  |  |  |  |  |
| Physical activity (step counts/min) | 0.90 (0.91) | 0.71 (0.61) | 0.39 (0.39) | 0.153 | 0.210 |
| Average minutes | 231.3 (8.8) | 232.8 (7.4) | 232.4 (9.6) | 0.936 | 0.983 |
| Average step counts | 205.3 (202.4) | 164.6 (138.7) | 89.9 (89.5) | 0.108 | 0.202 |
| **Early morning (03:00-07:00)** |  |  |  |  |  |
| Physical activity (step counts/min) | 0.66 (0.69) | 0.40 (0.31) | 1.25 (1.32) | 0.175 | 0.550 |
| Average minutes | 231.4 (6.3) | 231.2 (5.0) | 221.3 (9.6) | **0.004** | **0.026** |
| Average step counts | 151.1 (156.3) | 93.0 (71.3) | 279.1 (299.7) | 0.194 | 0.576 |

PCC patients were clinically divided into groups of increased or normal sympathetic activity based on self-reported symptoms. Self-Reported Symp-PCC refers to PCC patients who reported symptoms suggestive of sympathicotonia (e.g. sensation of high resting heart rate, palpitations, or sleep disturbances), while Self-Reported Non-Symp-PCC refers to PCC patients who did not report such symptoms. Healthy, non-symptomatic control subjects are included for comparison.

Table S2. Orthostatic test results, medication use, and DNE recording quality data for groups, classified based on orthostatic sympathetic response (Ortho-Symp and Ortho-Non-Symp).

| **All participants with both orthostatic test and EDA-measurement completed, N=35** | Ortho-Non-Symp  n = 14 | Ortho-Symp  n = 21 | Non-adjusted | Adjusted for age, sex and BMI^†^ |
| --- | --- | --- | --- | --- |
|  | Mean (SD) | | p | p |
|  |  |  |  |  |
| **Orthostatic test results** |  |  |  |  |
| HR rest | 73.6 (15.2) | 66.0 (9.0) | 0.072 | 0.079 |
| HR immediately | 96.4 (18.4) | 101.2 (10.5) | 0.338 | 0.868 |
| Increase of HR | 22.9 (7.0) | 35.2 (7.5) | **<0.001** | **0.001** |
| HR 1 min | 82.3 (18.1) | 81.4 (12.0) | 0.823 | 0.501 |
| HR 8 min | 92.2 (33.4) | 84.4 (14.4) | 0.350 | 0.137 |
| Blood pressure (systolic) rest | 132.8 (15.4) | 116.1 (11.6) | **0.002** | **0.011** |
| Blood pressure (systolic) immediately | 137.1 (18.3) | 119.6 (17.5) | **0.017** | **0.019** |
| Blood pressure (systolic) increase | 3.7 (12.0) | 4.2 (16.2) | 0.926 | 0.826 |
| Blood pressure (systolic) 1 min | 142.6 (20.1) | 125.9 (19.1) | **0.020** | 0.093 |
| Blood pressure (systolic) 8 min | 139.9 (21.3) | 118.6 (17.2) | **0.002** | **0.031** |
|  |  |  |  |  |
| **Medication** |  |  |  |  |
| Asthma medication | 3 | 3 |  |  |
| Allergy medication |  | 3 |  |  |
| Antihypertensive medication | 1 |  |  |  |
| Beta blockers | 2 |  |  |  |
| Hormone replacement therapy or contraceptives | 3 | 5 |  |  |
| Tranquilizers | 4 | 4 |  |  |
| Sleeping pills | 1 |  |  |  |
| Melatonin | 1 | 2 |  |  |
| Hypercholesterolemia medication | 3 |  |  |  |
| Anti-inflammatory medication |  | 1 |  |  |
|  |  |  |  |  |
| **DNE recording quality** |  |  |  |  |
| **Full day and night period** |  |  |  |  |
| Number of days | 11.8 (1.9) | 10.7 (2.8) | 0.219 | 0.706 |
| Physical activity (step counts/min) | 4.2 (1.0) | 3.6 (1.3) | 0.153 | 0.175 |
| Average minutes | 1286.1 (56.6) | 1283.1 (82.7) | 0.907 | 0.922 |
| Average step counts | 5384.8 (1323) | 4598.5 (1831) | 0.176 | 0.212 |
| **Night period (23:00-07:00)** |  |  |  |  |
| Physical activity (step counts/min) | 0.92 (0.74) | 0.66 (0.60) | 0.260 | 0.704 |
| Average minutes | 459.6 (10.5) | 457.4 (16.9) | 0.665 | 0.381 |
| Average step counts | 422.4 (345) | 299.8 (276) | 0.252 | 0.694 |
| **Day period (07:00-23:00)** |  |  |  |  |
| Physical activity (step counts/min) | 6.0 (1.4) | 5.1 (2.1) | 0.206 | 0.222 |
| Average minutes | 826.3 (10.5) | 825.7 (77.7) | 0.980 | 0.770 |
| Average step counts | 4962.3 (1161) | 4298.9 (1829) | 0.238 | 0.222 |
| **Late night (23:00-03:00)** |  |  |  |  |
| Physical activity (step counts/min) | 0.57 (0.64) | 0.64 (0.70) | 0.746 | 0.703 |
| Average minutes | 233.6 (8.1) | 231.1 (9.4) | 0.426 | 0.483 |
| Average step counts | 131.1 (148.8) | 146.2 (153.7) | 0.775 | 0.720 |
| **Early morning (03:00-07:00)** |  |  |  |  |
| Physical activity (step counts/min) | 1.29 (1.32) | 0.68 (0.81) | 0.098 | 0.455 |
| Average minutes | 226.0 (7.3) | 226.3 (10.7) | 0.460 | 0.489 |
| Average step counts | 291.3 (300.2) | 153.5 (184.6) | 0.101 | 0.453 |

^†^adjusted for age, sex and BMI

Table S3. Baseline characteristics, medication use, DNE values, and DNE recording quality data for PCC patients and controls divided into four groups, based on orthostatic sympathetic response (Ortho-Non-Symp-Control, Ortho-Symp-Control, Ortho-Non-Symp-PCC, Ortho-Symp-PCC).

|  | Ortho-controls | | Ortho-PCC | |  |  |
| --- | --- | --- | --- | --- | --- | --- |
|  | Ortho-Non-Symp-Cont N=7 | Ortho-Symp-Cont  N=11 | Ortho-Non-Symp-PCC  N=7 | Ortho-Symp-PCC  N=10 | ANOVA  p-value | adjusted p-value (age, sex, BMI) |
| Variable | Mean (SD) | Mean (SD) | Mean (SD) | Mean (SD) |  |  |
|  |  |  |  |  |  |  |
| Age (y) | 51.6 (12.4) | 47.5 (10.1) | 44.9 (8.2) | 38.2 (9.0) | 0.141 | - |
| BMI (kg/m2) | 28.6 (4.6) | 25.7 (4.0) | 28.6 (3.6) | 23.8 (2.9) | 0.013 | - |
| Sex, male/female | 1/6 | 2/9 | 3/4 | 4/6 | 0.454 | - |
| **Medication** |  |  |  |  |  |  |
| Asthma medication | 1 |  | 2 | 3 |  |  |
| Allergy medication |  |  |  | 3 |  |  |
| Antihypertensive medication | 1 |  |  |  |  |  |
| Beta blockers |  |  | 2 |  |  |  |
| Hormone replacement therapy or contraceptives | 2 | 2 | 1 | 3 |  |  |
| Tranquilizers | 2 | 1 | 2 | 3 |  |  |
| Sleeping pills |  |  | 1 |  |  |  |
| Melatonin |  |  | 1 | 2 |  |  |
| Hypercholesterolemia medication | 1 |  | 2 |  |  |  |
| Anti-inflammatory medication |  |  |  | 1 |  |  |
|  |  |  |  |  |  |  |
| **DNE and recording quality** |  |  |  |  |  |  |
| **Whole day and night period** |  |  |  |  |  |  |
| DNE | 52.8 (4.6) | 51.8 (3.2) | 51.2 (2.0) | 50.9 (12.3) | 0.880 | 0.856 |
| Number of days | 10.1 (2.3) | 8.3 (2.5) | 11.0 (1.5) | 11.0 (2.3) | **0.033** | **0.030** |
| Physical activity | 4.2 (1.2) | 3.5 (1.2) | 4.1 (0.8) | 3.7 (1.5) | 0.525 | 0.493 |
| Average minutes | 1270.6 (65.4) | 1240.6 (78.3) | 1301.6 (45.9) | 1329.8 (61.2) | **0.027** | **0.034** |
| Average step counts | 5418.6 (1571.1) | 4306.1 (1148.6) | 5351.0(1148.6) | 4920.2 (2088.1) | 0.480 | 0.401 |
| **Night period (23:00-07:00)** |  |  |  |  |  |  |
| DNE level | 39.1 (9.9) | 40.1 (9.5) | 30.5 (9.0) | 39.4 (17.0) | 0.380 | 0.289 |
| Physical activity | 1.15 (0.91) | 0.59 (0.52) | 0.69 (0.51) | 0.71 (0.71) | 0.366 | 0.490 |
| Average minutes | 458.1(11.4) | 466.1 (11.1) | 461.1 (10.2) | 449.0 (17.1) | 0.111 | 0.075 |
| Average step counts | 529.1 (418.3) | 267.1 (245.3) | 315.6 (238.4) | 335.7 (315.2) | 0.359 | 0.482 |
| **Day period (07:00-23:00)** |  |  |  |  |  |  |
| DNE level | 61.1 (5.0) | 58.6 (8.0) | 62.7 (5.8) | 56.1 (14.5) | 0.538 | 0.243 |
| Physical activity | 6.1 (1.7) | 5.1 (1.8) | 6.0 (1.2) | 5.3 (2.4) | 0.655 | 0.539 |
| Average minutes | 809.1 (61.2) | 791.8 (81.9) | 843.4 (44.0) | 862.9 (54.9) | 0.081 | 0.082 |
| Average step counts | 4889.4 (1337.3) | 4039.1 (1571.1) | 5035.1 (1057.6) | 4584.5 (2124.7) | 0.580 | 0.437 |
| **Late night (23:00-03:00)** |  |  |  |  |  |  |
| DNE level | 33.4 (11.8) | 40.4 (9.2) | 32.2 (7.8) | 41.2 (16.9) | 0.313 | 0.256 |
| Physical activity | 0.38 (0.56) | 0.39 (0.26) | 0.75 (0.70) | 0.92 (0.92) | 0.222 | 0.299 |
| Average minutes | 236.3 (6.7) | 229.9 (10.6) | 230.9 (8.8) | 232.4 (8.2) | 0.515 | 0.579 |
| Average step counts | 89.7 (128.1) | 90.1(61.5) | 172.4 (166.1) | 207.9 (200.6) | 0.225 | 0.579 |
| **Early morning (03:00-07:00)** |  |  |  |  |  |  |
| DNE level | 45.2 (8.8) | 39.8 (10.9) | 28.8 (11.8) | 37.7 (18.1) | 0.154 | 0.190 |
| Physical activity | 1.97 (1.6) | 0.80 (0.91) | 0.63 (0.48) | 0.55 (0.71) | 0.030 | 0.171 |
| Average minutes | 224.9 (10.0) | 219.1 (9.0) | 227.1 (3.5) | 234.3 (5.3) | 0.225 | 0.293 |
| Average step counts | 439.6 (363.4) | 176.9 (209.7) | 143.0 (109.1) | 127.7 (159.4) | 0.035 | 0.183 |

DNE = a double normalized index of electrodermal activity; Full day period DNE level = the average of daily DNE values across all 14 days; Night period (23:00-07:00) DNE level = the average of DNE values between 23:00 and 07:00 across all 14 days; Day period (07:00-23:00) DNE level = the average of DNE values between 07:00 and 23:00 across all 14 days; Late night (23:00-03:00) DNE level = the average of DNE values between 23:00 and 03:00 across all 14 days; Early morning (03:00-07:00) DNE level = the average of DNE values between 03:00 and 07:00 across all 14 days.
